# Roles of Mitochondrial Dynamics under Stressful and Normal Conditions in Yeast Cells

**DOI:** 10.1155/2013/139491

**Published:** 2013-07-14

**Authors:** Dmitry A. Knorre, Konstantin Y. Popadin, Svyatoslav S. Sokolov, Fedor F. Severin

**Affiliations:** ^1^Belozersky Institute of Physico-Chemical Biology, Moscow State University, Vorobyevy Gory 1, Moscow 119992, Russia; ^2^Institute of Mitoengineering, Moscow State University, Vorobyevy Gory 1, Moscow 119992, Russia; ^3^Department of Genetic Medicine and Development, University of Geneva Medical School, 1 rue Michel-Servet, 1211 Geneva, Switzerland

## Abstract

Eukaryotic cells contain dynamic mitochondrial filaments: they fuse and divide. Here we summarize data on the protein machinery driving mitochondrial dynamics in yeast and also discuss the factors that affect the fusion-fission balance. Fission is a general stress response of cells, and in the case of yeast this response appears to be prosurvival. At the same time, even under normal conditions yeast mitochondria undergo continuous cycles of fusion and fission. This seems to be a futile cycle and also expensive from the energy point of view. Why does it exist? Benefits might be the same as in the case of sexual reproduction. Indeed, mixing and separating of mitochondrial content allows mitochondrial DNA to segregate and recombine randomly, leading to high variation in the numbers of mutations per individual mitochondrion. This opens a possibility for effective purifying selection-elimination of mitochondria highly contaminated by deleterious mutations. The beneficial action presumes a mechanism for removal of defective mitochondria. We argue that selective mitochondrial autophagy or asymmetrical distribution of mitochondria during cell division could be at the core of such mechanism.

## 1. Introduction 

Almost all eukaryotic cells harbor mitochondria—the distant descendants of endosymbiotic alphaproteobacteria (see [1] for review). In most types of eukaryotic cells, mitochondria are organized in complex reticular structures with dynamically changing topology. Changes of the structures are usually referred to as mitochondrial dynamics. These changes include events of mitochondria fission and fusion, as well as active transport of individual filaments. Importantly, defects of mitochondrial dynamics are associated with defects in mechanisms of mitochondrial DNA (mtDNA) maintenance [2]. This makes the baker's yeast *Saccharomyces cerevisiae *a useful tool for studies of mitochondrial dynamics mechanisms. Importantly, in contrast to most of animal cells, yeasts are able to proliferate without a functional respiratory chain and ATP-synthase. Such cells still have basal mitochondrial morphology, membrane potential, and metabolic activities. Moreover, yeast cells lacking mitochondrial DNA can be easily cultivated in medium containing a fermentable carbon source (e.g., glucose). This allowed performing several large-scale screenings aimed at identification of genes involved in mitochondrial dynamics and maintenance of reticular mitochondrial morphology [2, 3]. It was found that proteins involved in the molecular mechanisms of mitochondrial fusion and fission in yeast have many similarities to such mechanisms in animals (see [4] for review). The molecular mechanisms of mitochondrial fusion, fission, and movement were reviewed in detail recently [4–8].

In this review we briefly discuss these mechanisms focusing on the role of mitochondrial dynamics in cell physiology under stressful and normal conditions using an example of *Saccharomyces cerevisiae* yeast cells. 

## 2. Mitochondrial Dynamics: Mechanisms and Stress Response

Mitochondrial fission is an active process organized by several proteins (see [9] for review). The key role is played by GTPase Dnm1p, which is capable of oligomerization on the lipid surface: it was shown that Dnm1p forms ~80 nm spirals on liposomes [10, 11]. A subsequent GTP hydrolysis results in a conformational change of Dnm1p, constriction of the spirals, and fission of the lipid vesicles [11]. Fis1p is localized at the outer membrane of mitochondria and recruits Dnm1p on the mitochondrial surface by means of adaptor proteins: Caf4p and its paralog Mdv1p [12]. It was shown that during membrane scission Dnm1p-dependent constriction is accompanied by longitudinal tension of lipid vesicles [13]. This implies that mitochondrial movement along the cytoskeleton could be an important factor for mitochondrial fission. Interestingly, for some stressful conditions fission of mitochondrial reticulum was observed even in the absence of the *DNM1 *gene. Kitagaki and colleagues [14] have shown that high (15%) concentration of ethanol facilitates fission of yeast mitochondria even on *Δdnm1* background. Possibly, severe changes in ion composition of cytoplasm and mitochondrial matrix or significant change in mitochondrial lipid composition leads to mechanical division of mitochondria into small compartments. Indeed, most of the protocols of mitochondria isolation do not allow preserving native structure of mitochondrial reticulum.

**Figure 1:**
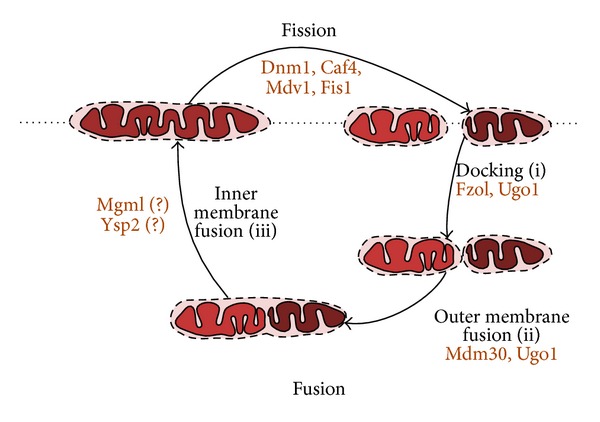
Proteins driving the cycle of mitochondrial fusion and fission. Mitochondrial fusion starts with the docking mediated by Fzo1 and Ugo1. Docking is followed by the outer membrane fusion mediated by Mdm30 and Ugo1. The inner membrane fusion is likely to be dependent on Mgm1 and Ysp2. Caf4, Mdv1 and Fis1 make a platform for fission which is being executed by Dnm1 oligomers.

 Mitochondrial fusion in yeast is orchestrated by another set of proteins including Fzo1p, Mdm30, Ugo1p, and Mgm1p. Studies on isolated mitochondria show that the process of fusion occurs in several stages [15, 16]: docking of mitochondria and fusion of the outer membranes followed by fusion of the inner membranes (Figure 1). Docking of mitochondria in yeast is controlled by outer membrane protein Fzo1p. Fzo1p is a GTPase [17] that forms trans-dimers allowing anchoring of separate mitochondrial vesicles. According to the model of Anton and colleagues [16], this process requires GTP binding, whereas hydrolysis of GTP allows moving to the next stage—fusion of the outer membranes. While mitochondrial fusion requires GTP, Meeusen and McCaffery [15] have shown that the rate of mitochondrial fusions could be as high as 50% of the maximum level with energy regeneration system in the absence of exogenous GTP. This indicates that low concentration of endogenous GTP is sufficient to maintain physiological rates of mitochondrial fusion. At the same time, the uncoupler FCCP (Carbonyl cyanide-4-(trifluoromethoxy)phenylhydrazone) and the ionophore valinomycin were found to be effective inhibitors of fusion of isolated yeast [15] and human [18] mitochondria. Accordingly, FCCP [19] and also other uncouplers and inhibitors of respiratory chain were found to induce mitochondrial reticulum fission in various human cell lines (see [20] for review). Another important factor regulating mitochondrial fusion is cellular redox state. Very recently, it was shown that docking of mitochondria is regulated by the ratio of reduced/oxidized glutathione: GSSG induces the generation of disulphide-mediated mitofusin oligomers and in this way promotes fusion [21]. It was found that C684A mutation of cysteine in Mfn2 inhibits mitochondrial fusion, pointing to a role of this residue in oligomerization of mitofusin and docking of mitochondria [22]. As *S. cerevisiae* genome contains a homolog of Mfn2, FZO1, the redox-dependent mechanism of regulation is likely to be conserved also in yeast. At the same time, treatment of human cells with hydrogen peroxide [22] or treatment of yeast cells with the drug amiodarone that induces endogenous formation of reactive oxygen species [23] causes mitochondrial fission. This apparent contradiction could be explained by the fact that high concentration of hydrogen peroxide inhibits the respiratory chain, thus counteracting the stimulating effect on mitochondria fusion [24]. Another explanation is based on the importance of the lipid composition for mitochondrial fusion; the inactivation of genes encoding enzymes of mitochondrial biosynthesis of lipids, phosphatidylethanolamine and cardiolipin (*PSD1* and *CRD1*, correspondingly), inhibits mitochondrial fusion [25, 26]. Therefore, peroxidation of cardiolipin induced by hydrogen peroxide could also retard mitochondrial fusion.

To conclude this part, it should be noted that stresses generally tip the mitochondrial dynamics towards fission. This is not surprising; local damage to fused mitochondria might depolarize the whole cellular mitochondrial content [27]. In other words, cells with fissed mitochondria seem to be more resistant to stresses; at least a minor fraction of the mitochondrial vesicles might be lucky to escape the damage. In theory, a cell with fragmented mitochondria is as resistant as the one with separated mitoplasts surrounded by an intact outer membrane. We have recently shown that such morphology can be observed in *Δysp2 *cells in stationary phase [28]. The *YSP2 *gene was previously discovered as a genetic factor that decreases resistance to amiodarone and acetic acid [29]. This is consistent with a possible role of Ysp2p in mitochondrial inner membrane fusion (Figure 1).

## 3. Role of Mitochondrial Dynamics under Normal Conditions

 It must be noted that mitochondrial fusion and fission not only happen under stress, but also constantly occur under normal conditions. This is true not only for yeast cells, but also for cells of higher organisms. Both processes consume energy. Thus the question arises about the biological role of these processes under stress-free conditions.

 It has been suggested that fusion-fission cycle can maintain high quality of mitochondrial protein components, keeping mitochondrial vesicles with low membrane potential out of the process of fusion into the mitochondrial reticulum and their consequent elimination by mitophagy (mitochondria-specific autophagy; see [30] for review). This filtering will keep the average quality of the mitochondrial respiratory chain on a constant level and work against ROS-induced protein damage but will not affect mtDNA quality if segregation of proteins and mtDNA is independent during fusion-fission process. However, since mtDNA is a subject to high mutation rate [31], the effective purifying selection which eliminates *de novo* deleterious mutations is required to maintain the quality of mtDNA. Thus, it has been proposed that the fusion-fission process can not only select mitochondria on the level of phenotypes (proteins), but also contribute to preferential elimination of mtDNA with deleterious mutations [32]. However, the functional link between mitochondrial phenotypes (protein components) and genotypes (mtDNA) is necessary for establishment of this kind of selection on a genetic level. For example, low mitochondrial membrane potential needs to be determined by deleterious mutations in mtDNA. There are two not mutually exclusive ways to develop such phenotype-genotype linkage. The first is purely mechanistic and based on passive membrane colocalization of mtDNA and proteins encoded by the mtDNA. Indeed, the inner membrane proteins were shown to have significantly lower mobility than proteins localized in the outer membrane [33]. Thus the local quality of the mitochondrial membrane is expected to be linked to the quality of the neighboring mtDNA attached to the membrane. This membrane linkage will lead to the formation of fissed mitochondria with correlated quality of phenotypes and genotypes, making a “fusion quality filter” effective in elimination of mtDNA with deleterious mutations (see [34] for review).

**Figure 2:**
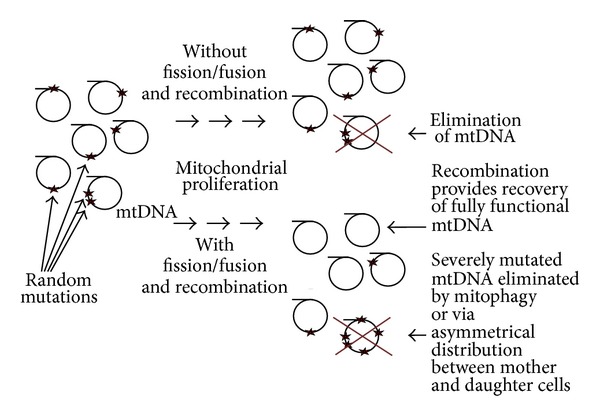
Hypothetical scheme illustrating how the mitochondrial fusion-fission cycle helps to maintain mitochondrial DNA. Rearrangements of the DNA induced by mitochondrial fission-fusion and recombination result in appearance of mutation-free mitochondrial genomes. Removal of genomes with high levels of deleterious mutations inhibits the high rate of mtDNA damage (see text for details).

 The second mechanism is based on active expression of mtDNA in fissed mitochondria and subsequent incorporation of newly synthesized proteins into mitochondrial membrane, so that genotype fitness influences phenotype fitness. This scenario does not depend on passive inheritance of mtDNA in complex with their protein products but requires long periods of existence of fissed mitochondria sufficient for incorporation of newly synthesized proteins into the mitochondrial membrane. The mechanism of purification of mitochondria from mutant copies of DNA during the fusion-fission process resembles the mechanism proposed to explain the existence of sexual reproduction (mutational deterministic hypothesis; [35, 36]). The fusion-fission process (as well as genetic recombination) restores high variability in the numbers of mutant mtDNAs per mitochondrial vesicle (number of deleterious mutations per offspring), making selection more effective (Figure 2). Indeed, elimination of the minimal numbers of mitochondrial vesicles maximally contaminated by mutant mtDNA will keep the heteroplasmy level low and will be energetically less expensive than elimination of large numbers of moderately contaminated mitochondria. The mutational deterministic hypothesis depends on high mutation rate and a negative epistasis between deleterious mutations [35–37]. Since both parameters are poorly known for the mitochondrial genome, future experimental studies are required to test the importance of the mechanism.

 The relative influence of the first (passive) and the second (active) mechanisms on the maintenance of the mtDNA quality during the fusion-fission process depends on the frequency of fusion-fission events and the rate of mtDNA transcription and translation, and thus they can be different in various cells.

 Is there any experimental evidence that mitochondrial dynamics counterbalance the high rate of deleterious mutation accumulation in yeast? It was recently shown that the *FIS1 *and *MDV1 *genes appear to be involved in maintenance of heteroplasmy in diploid yeast cells acquired after mating of haploids cells with different mitochondrial markers [38]. Although the authors did not observe this effect in *Δdnm1/Δdnm1* diploid, the results indicated that mitochondrial dynamics affect redistribution of different variants of the mitochondrial genome. The knockouts of genes involved in mitochondrial dynamics increase the chance of *petite* mutation in yeast. *Petite* mutation refers to inability of growth on medium with a nonfermentable carbon source (ethanol, glycerol, etc.), and it is usually associated with deletions or complete loss of mtDNA (see [39] for review). The inactivation of a fusion protein-encoding gene (*FZO1*) results in quick degradation of the mitochondrial genome [40], whereas the deletions of fission genes *DNM1* or *FIS1*, while increasing rate of petite mutation, still allow maintenance of the functional copies of mtDNA in the cells.

 To draw parallels between sexual reproduction and fusion-fission dependent recombination of mtDNAs one should consider the ways of elimination of nonfunctional mitochondria. Apparently, the functional mtDNA could outcompete the damaged one by advantages in replication. Although the opposite situation is described in the literature as suppressive petite mutation [41], the latter is not a common case. An alternative explanation comes from experiments with human cell lines. In 2008, Twig and colleagues [42] showed that after fission mitochondria can depolarize and get targeted to an autophagosome. The process of selective elimination of mitochondria unable to maintain their own transmembrane potential was called mitophagy. Mitophagy attracted a lot of attention because the mutations in PINK1 and Parkin (involved in marking the malfunctioning mitochondria for mitophagy) were found to be linked to Parkinson disease (see [43, 44] for review). It was suggested that mitophagy is necessary for quality control of mitochondria, to remove damaged protein complexes [8] or damaged mtDNA [32]. There are no homologs of PINK1 or Parkin in *Saccharomyces cerevisiae. *However, certain conditions, including nitrogen starvation or rapamycin treatment, induce selective mitochondrial autophagy in Atg32-dependent manner. Atg32 is located on the outer mitochondrial membrane and initiates the assembly of the autophagic complex which includes several ubiquitin ligases and adaptor proteins [45, 46]. Moreover, treatment of cells with mitochondrial respiratory chain inhibitors Antimycin A or KCN triggers a general increase of autophagy [47]. Intriguingly, this increase was shown to be Atg32 dependent [47]. Recently, it was shown that mitophagy indeed plays a role in maintaining mitochondrial quality in yeast [48]. 

Interestingly, it was shown that mitochondrial fission is not a necessary prerequisite for mitophagy in yeast [49]. This underscores the importance the “futile” fission-fusion cycle; the mitophagy of fully fused mitochondria can be lethal for the cells (yeast cannot live without mitochondria).

 Another possible way to remove mtDNA with deleterious mutations relies on asymmetrical division of yeast cells (see [50] for review). It was shown that a complex molecular mechanism (based on the polarisome) fulfills bud-to-mother transport of damaged macromolecules in yeast cells [51]. Moreover, it was recently reported that mitochondria with higher rate of superoxide production are retained in the mother cells [52]. Such selective retention is not surprising because actin cables anchor mitochondria via Mmm1p and Mdm10p [53], which in turn form a protein complex spanning both mitochondrial membranes and being capable of binding mtDNA (see [54] for review). The machinery responsible for mother-bud distribution of mitochondria also includes mitochondrial anchor in the mother cell (cortical protein Num1 [55] and myosin-related motor which moves mitochondria towards the bud (Myo2, [56]). Possibly, multiple rounds of asymmetrical distribution of malfunctioning mitochondria lead to a decrease of mtDNA stability in replicatively old cells. In accordance with this hypothesis, it was shown that the chances of *petite *mutation significantly increased with replicative age [57].

 Replicatively old yeast cells demonstrate slower growth and increased chance of viability loss [58]. Some authors argue that the death of a replicatively old mother cell could be an active process similar to apoptosis of mammalian cells [59]. Therefore, if asymmetrical redistribution of mitochondria indeed exists, damaged mtDNA is constantly eliminated by replicative aging.

 Is there any evidence linking yeast replicative aging and mitochondrial dynamics? Recently it was found that replicatively old yeast cells harbor fissed mitochondria with decreased transmembrane potential [60]. Earlier Scheckhuber and colleagues [61] showed that replicatively aged yeast cells have fissed mitochondria and that the deletions of either *DNM1* or *FIS1* genes provide a certain increase in *S. cerevisiae *lifespan. Indeed, mitochondrial dynamics appear to be necessary for asymmetrical redistribution of mitochondria. Theoretically, if the dynamics are lost due to mutations, then the old mother cells of the mutants gain some advantage over the wild type ones carrying accumulating copies of damaged mtDNA. However, this benefit is obviously canceled by a moderate decrease in competitive fitness of the mutant cells; high-throughput screening [62] has shown a decrease in competitive fitness of strains lacking* FIS1, CAF4, DNM1,* or *FZO1* compared to the wild type strain.

## 4. Conclusions 

 To conclude, it appears that mitochondrial dynamics in yeast play different roles under stress and under normal conditions and normal aging. A single onset of fission followed by recovery of the reticulum seems to be required for the stress-resistances. Alternatively, multiple “futile cycles” of fusion and fission contribute to maintenance of mitochondrial genomes in the long term under stress-free conditions.
